# Editorial: Mechanics of Cell Division

**DOI:** 10.3389/fcell.2020.620111

**Published:** 2020-11-30

**Authors:** Yuta Shimamoto, Stefanie Redemann, Daniel Needleman

**Affiliations:** ^1^Physics and Cell Biology Laboratory, Department of Chromosome Science, National Institute of Genetics, Shizuoka, Japan; ^2^Department of Genetics, SOKENDAI University, Shizuoka, Japan; ^3^Department of Molecular Physiology and Biological Physics, Center for Membrane and Cell Physiology, School of Medicine, University of Virginia, Charlottesville, VA, United States; ^4^Department of Cell Biology, School of Medicine, University of Virginia, Charlottesville, VA, United States; ^5^Department of Molecular and Cellular Biology and School of Engineering and Applied Sciences, Harvard University, Cambridge, MA, United States; ^6^Center for Computational Biology, Flatiron Institute, New York, NY, United States

**Keywords:** mitosis, mitotic spindle apparatus, chromosome segregation, cytoskeleton, motor protein, kinetochore, mechanical force

Cells proliferate and differentiate through division. At each cell division, replicated chromosomes are equally partitioned into the newly-created two daughter cells. Missegregation of chromosomes can lead to aneuploidy—the hallmark of cancer, infertility, and several developmental disorders such as Down's syndrome. Cells use multitude mechanisms to ensure accurate cell division. Understanding the molecular and mechanical basis of accurate chromosome segregation should help develop strategies to control cell division and to cure and prevent diseases associated with chromosome missegregation.

Cell division involves diverse forces and motions, which take place in precise temporal and spatial orders. When cells enter mitosis, the nuclear envelope breaks down and chromosomes are organized into a rigid, rod-like shape by actions of chromosome-associated proteins. These chromosomes are then captured by the bipolar spindle—the dynamic cytoskeletal structure assembled from microtubules and microtubule-associated proteins. Microtubules in the spindles are organized by molecular motors (i.e., dynein and kinesins) which generate forces between microtubules and cause them to slide relative to each other, and by diverse proteins that modulate microtubule nucleation, growing, and shrinking dynamics. Once chromosomes are captured, they are transported toward the center of the spindle, where they are aligned on the metaphase plate. Errors that arise associated with the chromosome-spindle attachment can result in missegregation of the pair of sister chromatids into one of the two daughter cells or can cause chromosomes to lag at the middle of the dividing cell. These mis-attachments are believed to be a major cause of chromosome segregation errors. Erroneous spindle-chromosome attachments are monitored and corrected by a complex machinery at the kinetochore—the multi-component structure assembled on centromeric chromatin that mediates microtubule binding to chromosomes. It is thought that the primary mechanism of error correction is based on the kinetochore sensing mechanical tension that arises across sister chromatids and controls its affinity for microtubules such that the correct, bioriented attachments are selectively stabilized. Once all chromosomes establish the correct spindle attachments, the linkage that holds sister chromatids is disengaged, leading to their physical separation toward the opposite ends of the cell. Finally, the partitioning of chromosomes is completed by the formation of a contractile ring, made from actin, the molecular motor myosin II, and other associated proteins. The constriction of the contractile ring between the separated chromosomes finally creates two daughter cells with identical genome copies. Together, the diverse types of force-generating activities co-operate in time and space to achieve successful cell division. In addition to such mechanical aspects of subcellular structures, the shape and mechanics of the entire cell itself also influence cell division. For example, interphase cells, which are often flat as they adhere onto substrates, become round when they enter mitosis. Failures in cell rounding can lead to chromosome segregation errors in tissues and 3-D organoids, and depends on the physical context at which cells locate (e.g., the adhesion strength, tissue stiffness). Taken together, cell division requires an orchestration of diverse mechanical events that take place inside and outside the cell. This article collection covers the most up-to-date knowledge on this matter, as highlighted below.

Many cellular motions are driven by active forces exerted by cytoskeletal motor proteins, such as kinesin, myosin, and dynein. Among the three types, dynein has a unique regulatory role distinct from other motors as dynein has only a single isoform while performing diverse intracellular functions in dividing cells. Torisawa and Kimura highlight how dynein achieves distinct functions for proper cell division, by focusing on varying types of regulations, such as those via intra-molecular mechanochemical coupling, through accessory proteins, and by external forces. Dynein can work as an ensemble to move cargoes and microtubules for the generation of cellular-scale dynamics. The authors introduce the process of meiotic cell division in *C. elegans*, and highlight its intricate spatial and temporal regulation in cell division.

The kinetochore is a proteinaceous structure assembled onto centromeric chromatin of mitotic chromosomes that mediates interactions with spindle microtubules. This structure exhibits multiple counterintuitive behaviors. First, it tracks the tips of microtubules that dynamically grow and shrink. Second, this dynamic attachment is mechanically stable enough to withstand a pulling force exerted by the thick microtubule bundle of the spindle, called the k-fiber. Finally, this attachment is regulated such that erroneous connections can be corrected. Wimbish and DeLuca review recent progresses in understanding the molecular architecture and mechanics of this attachment regulation in the kinetochore, particularly by focusing on the NDC80 complex, a heterotetrametric protein assembly comprised of Hec1, Nuf2, Spc24, and Spc25. The authors discuss how the N-terminal tail of Hec1 controls kinetochore's attachment stability to microtubules, via a layer of mechanisms such as phosphorylation and self-oligomerization.

Forces generated by motor proteins push and pull cytoskeletal filaments in a dividing cell. A large body of studies has focused on forces exerted at the cell cortex. For example, the mechanism in which dynein localizes to the cell cortex and pulls astral microtubules to control spindle position in the cell has been widely studied. Xie and Minc highlight another important mechanism of cellular force generation—the force generated in the bulk cytoplasm. This type of force originates from self-organizing cytoskeletal networks, which can either contract or expand to drive large-scale fluid flow in the cytoplasm and move micron-sized objects such as organelles in cells. The authors discuss detailed force-generating mechanisms and also introduce tools to measure and perturb these bulk cytoplasmic forces.

When cells enter mitosis, they often detach from the substrate and become round. The rounding creates a space for spindle assembly in the cell, and its failure can lead to chromosome segregation errors. Taubenberger et al., review the micromechanics associated with this dynamic structural change that takes place in mitotic cells. The authors discuss that cell rounding is not solely driven by passive forces due to the liquid-like nature of cells, but requires active forces that are generated by cortical tension and intracellular pressure, which both increase in mitosis. The molecular mechanism underlying the active force generation involves diverse processes including actomyosin contractility, the recruitment of intermediate filaments, and water influx. Cell rounding may have important functions in the context of tissues and developing embryos. The possible mechanisms and impacts on developmental processes are also discussed.

Recent advancement of computer technologies highlights the use of machine learning and artificial intelligence to analyze cell morphology and dynamics. Techniques developed are useful for studying a variety of intracellular events, including the mitotic dynamics of budding yeast—a widely used model for eukaryotic cell division. Lawrimore et al., developed a sophisticated analysis pipeline that allows them to automatically classify experimentally-obtained fluorescence images that pinpoint the location of kinetochore proteins in cells. The information is then used to build a model describing the nanoscale architecture of this multi-protein complex. The authors also demonstrate the use of their image analysis pipeline for nucleoli—the organelle that forms around a specific locus of the genome in the nucleus. The results show the versatility of their approach to forward-modeling the detailed structural architectures of biological assemblies.

## Author Contributions

YS, SR, and DN wrote the manuscript. All authors contributed to the article and approved the submitted version.

## Conflict of Interest

The authors declare that the research was conducted in the absence of any commercial or financial relationships that could be construed as a potential conflict of interest.

